# Lipid Flippase Subunit Cdc50 Mediates Drug Resistance and Virulence in *Cryptococcus neoformans*

**DOI:** 10.1128/mBio.00478-16

**Published:** 2016-05-10

**Authors:** Wei Huang, Guojian Liao, Gregory M. Baker, Yina Wang, Richard Lau, Padmaja Paderu, David S. Perlin, Chaoyang Xue

**Affiliations:** a Public Health Research Institute Center, New Jersey Medical School, Rutgers University, Newark, New Jersey, USA; b Department of Microbiology, Biochemistry and Molecular Genetics, New Jersey Medical School, Rutgers University, Newark, New Jersey, USA; c School of Pharmaceutical Sciences, Southwest University, Chongqing, China

## Abstract

*Cryptococcus neoformans* is a human fungal pathogen and a major cause of fungal meningitis in immunocompromised individuals. Treatment options for cryptococcosis are limited. Of the two major antifungal drug classes, azoles are active against *C. neoformans* but exert a fungistatic effect, necessitating long treatment regimens and leaving open an avenue for emergence of azole resistance. Drugs of the echinocandin class, which target the glucan synthase and are fungicidal against a number of other fungal pathogens, such as *Candida* species, are ineffective against *C. neoformans*. Despite the sensitivity of the target enzyme to the drug, the reasons for the innate resistance of *C. neoformans* to echinocandins remain unknown. To understand the mechanism of echinocandin resistance in *C. neoformans*, we screened gene disruption and gene deletion libraries for mutants sensitive to the echinocandin-class drug caspofungin and identified a mutation of *CDC50*, which encodes the β-subunit of membrane lipid flippase. We found that the Cdc50 protein localized to membranes and that its absence led to plasma membrane defects and enhanced caspofungin penetration into the cell, potentially explaining the increased caspofungin sensitivity. Loss of *CDC50* also led to hypersensitivity to the azole-class drug fluconazole. Interestingly, in addition to functioning in drug resistance, *CDC50* was also essential for fungal resistance to macrophage killing and for virulence in a murine model of cryptococcosis. Furthermore, the surface of *cdc50Δ* cells contained increased levels of phosphatidylserine, which has been proposed to act as a macrophage recognition signal. Together, these results reveal a previously unappreciated role of membrane lipid flippase in *C. neoformans* drug resistance and virulence.

## IMPORTANCE

*Cryptococcus neoformans* is a fungal pathogen that is the most common cause of fungal meningitis, causing over 620,000 deaths annually. The treatment options for cryptococcosis are very limited. The most commonly used drugs are either fungistatic (azoles) or highly toxic (amphotericin B). Echinocandins are the newest fungicidal drug class that works well in treating candidiasis and aspergillosis, yet they are ineffective in treating cryptococcosis. In this study, we showed that the regulatory subunit of the lipid translocase (flippase), a protein that regulates the asymmetrical orientation of membrane lipids, is required for *C. neoformans* resistance to caspofungin, as well as for virulence during infection. This discovery identifies lipid flippase as a potential *C. neoformans* drug target, which plays an important role in the innate resistance of *C. neoformans* to echinocandins and in fungal virulence.

## INTRODUCTION

*Cryptococcus neoformans* is an opportunistic fungal pathogen that can infect the central nervous system (CNS) in immunocompromised individuals to cause life-threatening cryptococcal meningitis (1, 2). *C. neoformans* expresses several classical virulence factors, including the ability to grow at body temperature and produce melanin and the polysaccharide capsule. These features protect the fungus against the hostile host environment and help it to evade the host immune response (3, 4). In addition, *C. neoformans* is a facultative intracellular organism that can survive and proliferate inside macrophages (5). The mechanisms underlying *C. neoformans*-host interactions and its ability to withstand the antimicrobial activity of macrophages are still very incompletely understood.

Treatment options for cryptococcosis are very limited. Currently, an acute infection is treated with amphotericin B or azoles in combination with flucytosine (6). However, both treatment courses have serious drawbacks. Amphotericin B binds not only fungal but also human sterols and thus causes toxic side effects. Azoles, which specifically target ergosterol biosynthesis, are better tolerated by patients but are fungistatic rather than fungicidal and often require a lifetime regimen of the drug. The fungistatic nature and long-term duration of these regimens increase the probability of the development of drug resistance by *C. neoformans* (7). Thus, new and more efficacious treatments are urgently needed to combat cryptococcosis.

The therapeutic challenge in developing antifungal agents is that both fungi and their mammalian hosts are eukaryotes and therefore contain similar cellular machinery. One major fungus-specific drug target is the cell wall. Echinocandins are the latest-generation antifungal drug class that targets the cell wall with fungicidal activity against several major fungal pathogens, including *Candida* and *Aspergillus* species (8, 9). The target of this new drug class is the β-1,3-glucan synthase, the essential enzyme to produce β-1,3-d-glucan, a major cell wall component. β-1,3-Glucan synthase is encoded by the *FKS* genes, which were first identified in *Saccharomyces cerevisiae* (10). In *C. albicans*, there are three copies of the Fks1 homologs (*FKS1/GSC1*, *GSL1*, and *GSL2*), and *FKS1* plays a predominant role (11). In *Aspergillus fumigatus* and *Aspergillus nidulans*, a single gene, *FKS1*, encodes the enzyme activity (12, 13). In *C. neoformans*, there is a single *FKS1* homolog (14). Although this gene is essential for survival in *C. neoformans* and purified β-glucan synthase from this fungus is strongly inhibited by echinocandin drugs *in vitro* (15), *C. neoformans* is naturally resistant to echinocandins, and the mechanism of resistance remains unknown.

To investigate the molecular basis of the inherent resistance of *C. neoformans* to echinocandins, we performed a high-throughput genetic screen for cryptococcal mutants that are sensitive to caspofungin, a drug of the echinocandin class. After screening over 7,000 mutants from a random mutagenesis library and 3,000 mutants from a gene deletion collection (16), we found that the homolog of the *S. cerevisiae CDC50* gene is required for echinocandin resistance in *C. neoformans*. *CDC50* encodes a β-subunit of lipid flippase, which is involved in membrane aminophospholipid translocation, cell surface receptor signal transduction, vacuole organization, and maintenance of the asymmetrical distribution of phospholipids on the bilayer lipid membrane (17). We found that in addition to mediating caspofungin resistance, *C. neoformans CDC50* was required for maintaining normal stress resistance and normal development of fungal virulence factors, overcoming the antifungal activity of macrophages, and developing cryptococcosis in the mouse model *in vivo*. Together, these results implicate the Cdc50-mediated lipid trafficking pathway in antifungal drug resistance and fungal pathogenesis in *C. neoformans*.

## RESULTS

### Screening for caspofungin-sensitive mutants.

**FIG 1:**
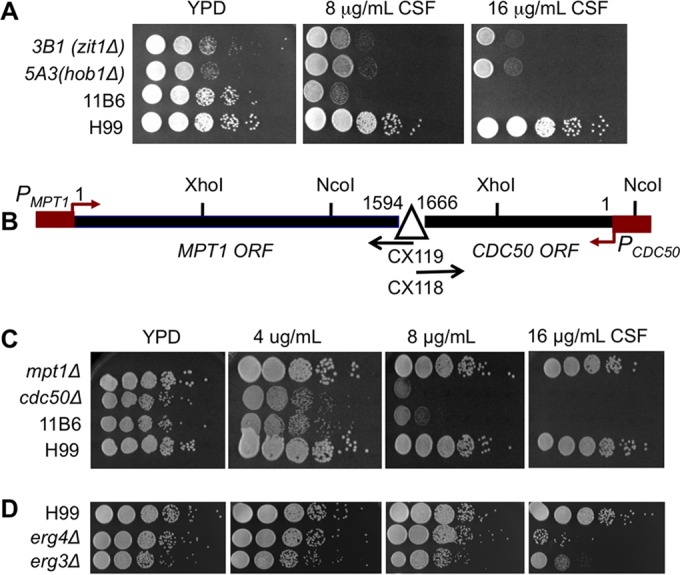
Identification of genes required for resistance to caspofungin. (A) Cultures of mutants sensitive to caspofungin (CSF) were grown overnight in YPD and diluted to an OD_600_ of 1.0. Tenfold serial dilutions were prepared, and 5 µl of each was spotted on YPD agar supplemented with 0, 8, or 16 µg/ml caspofungin. (B) Genomic location of T-DNA insertion marker in the mutant 11B6. Arrows indicate the direction of gene transcription. (C) Caspofungin sensitivity of deletion mutants of *MPT1* and *CDC50* that were identified from 11B6 by inverse PCR. (D) Caspofungin-sensitive mutants identified from the *C. neoformans* deletion library.

Using the wild-type strain H99, we generated an *Agrobacterium*-mediated mutagenesis library with ~7,000 transformants. This library was screened for mutants sensitive to caspofungin at 8 µg/ml in 96-well plates on yeast extract-peptone-dextrose (YPD) medium at 30°C. We identified three transformants (3B1, 5A3, and 11B6) that were sensitive to caspofungin but grew normally in its absence (Fig. 1). To confirm that the caspofungin-sensitive phenotype was linked with nourseothricin (NAT) resistance, the insertion marker, we performed a cosegregation assay by back-crossing these three transformants to the wild-type strain KN99**a**. In every case, caspofungin resistance segregated with NAT resistance. Next, to identify the sequences flanking the NAT marker and thus the disrupted genes, we used an inverse PCR method (18). Isolate 3B1 had a single insertion in gene *ZIT1*, which encodes an ion transporter homolog, while 5A3 had a single insertion in *HOB1*, which encodes an actin-associated protein with roles in endocytosis and exocytosis. The insertion in isolate 6B11 disrupted two adjacent genes: *CDC50*, which encodes a membrane protein homologous to Cdc50/Lem3 in *S. cerevisiae*, and *MPT1*, which encodes a mitogen-activated protein (MAP) kinase phosphatase. To determine which of these two disruptions was responsible for the phenotype in 11B6, we generated gene deletions for both *CDC50* and *MPT1*. We found that only the *cdc50Δ* null mutant, but not the *mpt1Δ* mutant, was sensitive to caspofungin (Fig. 1).

Besides the random mutagenesis library, we also screened two *C. neoformans* gene deletion collections generated by Hiten Madhani’s group at University of California San Francisco (UCSF) (16). A total of ~3,300 mutants were screened on YPD with 8 µg/ml caspofungin as described above. We identified two mutants (5F6 and 6A3) that were sensitive to caspofungin at this concentration (Fig. 1). Both of them are mutants of genes involved in the late steps of the ergosterol biosynthetic pathway: 5F6 is a null mutant of sterol C-24 reductase (Erg4; CNAG_02830), which catalyzes the last step of the ergosterol biosynthetic pathway, while 6A3 is a null mutant of sterol C-5 desaturase (Erg3; CNAG_00519), also involved in the late steps of the ergosterol biosynthesis. Thus, the two screens identified five genes potentially involved in mediating echinocandin resistance: *CDC50*, *ZIT1*, *HOB1*, *ERG4*, and *ERG3*. Among them, strains disrupting the *ZIT1* or *HOB1* gene also showed slow growth on YPD without drug. Because the *cdc50Δ* mutant exhibited the most pronounced sensitivity to caspofungin, we focused our further study on this gene.

### Cdc50 is required for caspofungin resistance.

**FIG 2:**
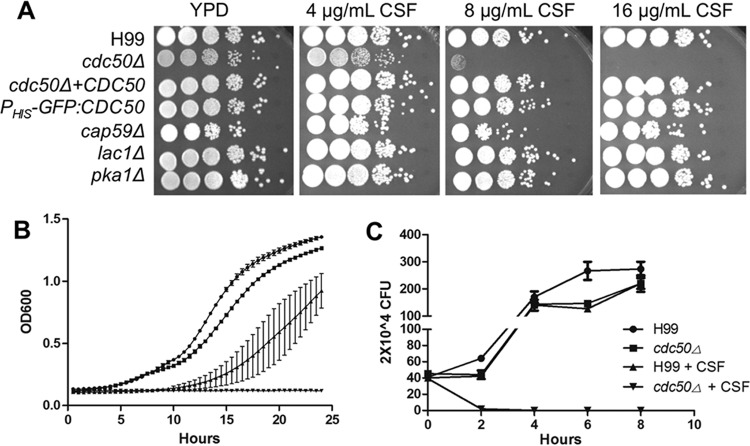
The *cdc50Δ* mutant is hypersensitive to caspofungin (CSF). (A) Cultures of indicated strains were grown overnight in YPD and diluted to an OD_600_ of 1.0. Tenfold serial dilutions were prepared, and 5 µl of each was spotted on YPD agar supplemented with 0, 4, 8, or 16 µg/ml caspofungin. (B) Of each strain, 1 × 10^5^ cells were inoculated into liquid YPD with or without 16 µg/ml caspofungin and incubated for 24 h at 30°C. Cell density was determined every 30 min by measuring OD_600_. (C) Survival rate of H99 and *cdc50Δ* strains on YPD with or without 16 µg/ml caspofungin. Yeast CFU were determined at different time points after incubation by plating onto drug-free medium.

To confirm the caspofungin sensitivity of the *cdc50Δ* mutant in *C. neoformans*, we deleted the entire *CDC50* open reading frame (ORF), replaced it with a NEO marker, and tested the resulting mutant as well as the complemented strain for growth in the presence and absence of caspofungin. We found that the *cdc50Δ* deletion mutant showed the same caspofungin sensitivity as the original insertion mutant and that drug resistance was restored by reintroducing the *CDC50* gene (Fig. 2A). Furthermore, we found that the *cdc50Δ* mutant was hypersensitive to caspofungin both on agar plates and in liquid medium (Fig. 2A and B). MIC testing showed that the MIC_90_ of the *cdc50Δ* mutant was >4-fold lower relative to wild-type strain H99 (4 µg/ml compared to over 16 µg/ml [Table 1]) following a 72-h incubation. Interestingly, the same mutant was also sensitive to another glucan synthase inhibitor, MK-3118, an enfumafungin derivative, but not to anidulafungin and micofungin, two other echinocandin-class drugs (Table 1). To determine whether caspofungin kills the *cdc50Δ* mutant or only inhibits its growth, we measured the viability of strains grown in liquid medium containing 16 µg/ml of caspofungin. Our results showed that fungal CFU of the *cdc50Δ* mutant decreased after drug treatment, indicating that caspofungin is fungicidal in the absence of *CDC50* (Fig. 2C).

**TABLE 1:** MICs on YPD at 30°C

Drug	MIC (µg/ml) of strain at 72 h on YPD		MIC range tested (µg/ml)
	H99	*cdc50Δ* mutant	
Anidulafungin	>64	>64	64–0
Caspofungin	16	4	64–0
Micafungin	>64	>64	64–0
MK-3118	4	2	64–0
Fluconazole	16	1	128–0
Voriconazole	0.25	0.01	8–0
Amphotericin B	2	0.5	8–0

Capsule and melanin are two unique features of cryptococci that are required for fungal virulence. To evaluate whether capsule or melanin is required for fungal resistance to echinocandin drugs, we examined mutants lacking these two virulence factors for their response to caspofungin (Fig. 2A). The *cap59Δ* mutant does not produce capsule (19), and the *lac1Δ* mutant shows a significant melanin production defect (20). We also used the *pka1Δ* mutant, which has a defect on both capsule and melanin production (21, 22). Our results showed that all three mutant strains had levels of drug resistance similar to that of the wild-type H99, indicating that neither capsule nor melanin is required for echinocandin resistance.

### Cdc50 prevents caspofungin uptake by *C. neoformans.*

**FIG 3:**
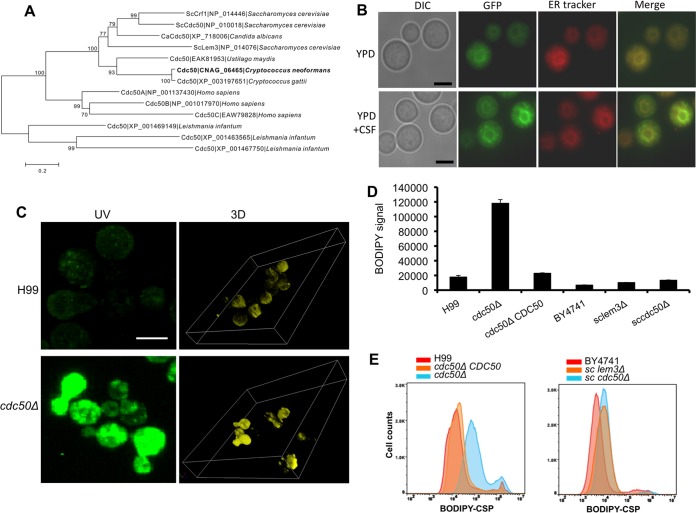
Loss of Cdc50 results in increased caspofungin uptake activity. (A) Phylogenetic analysis of Cdc50 homologs in different eukaryotes. The protein sequences were aligned with CLUSTAL X, and a neighbor-joining tree was generated using a Dayhoff model, 1,000 bootstrap replicates, and a pairwise deletion for gaps/missing data. (B) Fluorescent signal generated by Cdc50-GFP in cells grown on YPD or YPD with 8 µg/ml caspofungin (CSF) was colocalized with ER-Tracker Red. Bar, 5 µm. DIC, differential inference contrast. (C) H99 and *cdc50Δ* cultures were coincubated with 5 µmol BODIPY-labeled caspofungin for 30 min at 30°C. The fluorescent signal of fungal cells was detected by confocal fluorescence microscopy. (D) Fluorescent signal intensity was quantified using flow cytometry for 100,000 yeast cells of H99, the *cdc50Δ* mutant, and its complemented strain (*cdc50Δ+CDC50*), as well as *S. cerevisiae* strain BY4742 and its *lem3Δ* and *cdc50Δ* mutants treated with 5 µM BODIPY-caspofungin for 30 min at 30°C. Triplicates were used for each measurement. (E) Representative images of fluorescent signal quantification results using flow cytometry.

The Cdc50 that we identified in *C. neoformans* is the only homolog of an *S. cerevisiae* CDC50 gene family that contains three members (Cdc50, Lem3, and Crf1) (Fig. 3A). These proteins interact with type IV P-type ATPase and regulate its lipid flippase function, which is necessary to create and maintain an asymmetrical distribution of phospholipids in the plasma membrane as well as the membranes of the late secretory pathway and the endocytic compartments (17, 23, 24). Both Cdc50 and Lem3 in *S. cerevisiae* are localized to the endoplasmic reticulum (ER) and endosomal and plasma membranes to regulate the translocation of aminophospholipids, such as phosphatidylserine (PS). To determine if Cdc50 in *C. neoformans* also functions in these processes, we first analyzed its subcellular localization by expressing a Cdc50-green fluorescent protein (GFP) fusion protein under the control of the histone H4 promoter in the *cdc50Δ* mutant background. The GFP-expressing strain fully complemented the drug sensitivity of the *cdc50Δ* mutant (Fig. 2). We observed that the Cdc50-GFP fluorescent signal was mostly concentrated in membranes and largely colocalized with ER-Tracker staining, indicating that Cdc50 is localized to the ER membrane and also to the plasma membrane (Fig. 3B). We also treated the cells with caspofungin and observed increased plasma membrane localization after drug treatment, which is consistent with our finding that Cdc50 is required for caspofungin drug resistance in *C. neoformans* (Fig. 3B).

Because loss of *CDC50* is expected to perturb membrane function, we hypothesized that the *cdc50Δ* mutant may exhibit enhanced sensitivity to caspofungin because it alters drug uptake into cells. To test this hypothesis, we monitored drug uptake by using boron dipyrromethene difluoride (BODIPY)-labeled caspofungin (25). We observed that while H99 failed to take up significant amounts of fluorescently labeled caspofungin, producing only limited intracellular fluorescence, the *cdc50Δ* mutant showed a significantly higher level of fluorescent signal, indicative of an increased intracellular drug concentration (Fig. 3C; see also Fig. S1A in the supplemental material). We quantified this fluorescent signal for ~100,000 cells using flow cytometry and found that the *cdc50Δ* mutant had a significantly increased BODIPY-caspofungin uptake and that this increase was reversed by complementing the mutant with wild-type *CDC50* (Fig. 3D and E). Because the *cdc50Δ* mutant is more sensitive to caspofungin, we checked that the observed phenotype was not due to increased cell death of *cdc50Δ* cells in the presence of BODIPY-caspofungin. We measured cell viability after a 30-min BODIPY-caspofungin treatment and found that this short treatment did not result in increased killing of the mutant cells compared to the wild-type strain (see Fig. S1B).

We also examined the potential role of Cdc50 homologs in *S. cerevisiae* in caspofungin internalization. *S. cerevisiae* wild-type strain BY4741 and its *LEM3* and *CDC50* deletion mutant strains (sc*lem3Δ* and sc*cdc50Δ*, respectively) were coincubated with BODIPY-caspofungin as described above, and the fluorescent signal was analyzed by flow cytometry and microscopic observation. Our data showed that the overall signal intensities among the three strains were similar: the fluorescence signals for both sc*lem3Δ* and sc*cdc50Δ* mutants were mostly concentrated on the cell surface, similar to that of the wild type (Fig. 3D and E; see also Fig. S1C in the supplemental material).

Together, these results demonstrate that the *cdc50Δ* mutant significantly increases the internalization of caspofungin in *C. neoformans*, suggesting that poor drug uptake in the wild-type fungus may be one mechanism of drug resistance in *C. neoformans*. Because the *S. cerevisiae* parental strain BY4741 and its sc*lem3Δ* and sc*cdc50Δ* mutants had similar drug binding activities and drug localizations in the cell, the increased internalization of caspofungin in the *cdc50Δ* mutant in *C. neoformans* may be specific to this fungus.

### Cdc50 is required for azole resistance.

**FIG 4:**
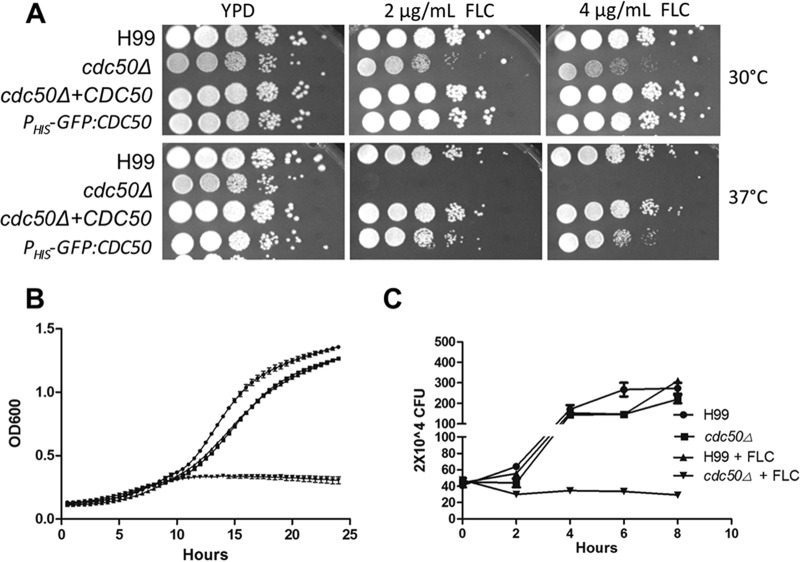
The *cdc50Δ* mutant is hypersensitive to fluconazole treatment. (A) Cultures of the (H99) *cdc50Δ* mutant and its complemented strains, as well as the *CDC50* overexpression strain, were incubated overnight in YPD and diluted to an OD_600_ of 1.0. Tenfold serial dilutions were prepared and plated on YPD containing 0, 2, and 4 µg/ml fluconazole (FLC) and incubated for 48 h at 30°C and 37°C. (B) Of each indicated strain, 1 × 10^5^ cells were inoculated in liquid YPD with or without 8 µg/ml fluconazole and grown for 24 h at 37°C. The OD_600_ of each culture was measured every 30 min. (C) Fungal survival rate was determined by CFU for H99 and *cdc50Δ* strains that were grown in liquid YPD with or without 8 µg/ml fluconazole.

As fluconazole is the first-line drug to treat cryptococcosis, we also investigated the role of *C. neoformans* CDC50 in resistance to azole drugs at both 30°C and 37°C. While fluconazole is fungistatic in *C. neoformans*, we used fluconazole concentrations (2 µg/ml and 4 µg/ml) that were not inhibitory to the growth of the wild-type strain on solid medium. The *cdc50Δ* mutant showed increased sensitivity to fluconazole at 37°C compared to the wild-type H99, while sensitivity was only moderately increased when the strain was incubated at 30°C (Fig. 4A). When the strains were grown in liquid YPD medium at 37°C in the absence or presence of 8 µg/ml fluconazole, the *cdc50Δ* mutant also showed much greater growth inhibition than did H99 (Fig. 4B and C). These results show that *C. neoformans* Cdc50 plays a broader role in fungal drug resistance.

### Cdc50 is required for membrane integrity.

**FIG 5:**
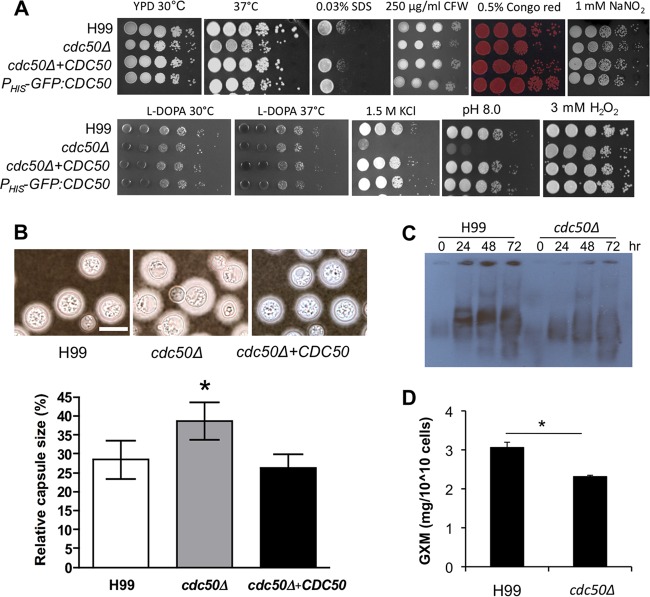
Cdc50 is involved in stress resistance and is required for normal development of classical *C. neoformans* virulence factors. (A) Cultures of H99, *cdc50Δ*, *cdc50Δ+CDC50*, and *CDC50* overexpression (*cdc50*Δ+*P_HIS_*-*GFP-CDC50*) strains were prepared with serial dilutions and were grown on YPD with 0.3% SDS, 250 µg/ml calcofluor white (CFW), 0.5% Congo red, 3 mM H_2_O_2_, 1 mM nitric oxide (NaNO_2_), or 1.5 M KCl. Cells were also grown on l-DOPA or YPD (pH 8.0) buffered with HEPES. The results were photographed after a 48-h incubation. Their growth on YPD or l-DOPA at 37°C was also determined by serial dilutions. (B) Capsule sizes of the above strains were determined by India ink staining after incubation on DME medium for 3 days at 37°C. Relative capsule size was determined by measuring over 50 cells for each strain. Bar, 10 µm; *, *P* < 0.01. (C) Electromobility of secreted GXM from H99 and the *cdc50Δ* mutant was detected in a Western blot assay as described in reference 50. Culture supernatants were collected at 0, 24, 48, and 72 h after inoculation and loaded on an 0.6% certified megabase agarose gel. The gel was transferred to a polyvinylidene difluoride membrane, and the GXM signal was detected using the GXM monoclonal antibody 18B7. (D) Quantification of secreted GXM from H99 and the *cdc50Δ* mutant. Secreted total polysaccharides were purified from a 500-ml YPD culture of each strain using the CTAB precipitation method as described previously (51). The amount of total GXM was determined by the phenol sulfuric method. Triplicates were used for each experiment. *, *P* < 0.01.

Next, we determined whether deleting *CDC50* affected cellular resistance to other types of stress, including cell wall and membrane stress, as well as oxidative, nitrosative, and osmotic stresses (Fig. 5A). No obvious change in sensitivity was observed in the presence of agents that disturb the cell wall—calcofluor white (CFW) and Congo red. However, the *cdc50Δ* cells were more sensitive to SDS, a phenotype that is usually associated with altered cellular membrane integrity. Cdc50 was not required for oxidative stress (3 mM H_2_O_2_) or nitrosative stress (1 mM NaNO_2_) resistance in our assays but did show increased sensitivity to osmotic stress (1.5 M KCl) and alkaline pH (Fig. 5A). Together, these data show that *C. neoformans* CDC50 is required for maintaining membrane integrity.

### Cdc50 is required for the development of fungal virulence factors.

To further probe the potential roles of Cdc50 in the pathogenesis of *C. neoformans*, we investigated whether its major virulence factors—ability to grow at 37°C and production of capsule and melanin—were affected in the *cdc50Δ* mutant. The *cdc50Δ* mutant grew normally at 30°C and exhibited a growth defect relative to wild-type and complemented strains at 37°C (Fig. 5A). The mutant also showed normal melanin production at both 30°C and 37°C. Interestingly, deletion of *CDC50* resulted in a significantly increased capsule size in cells grown on Dulbecco modified Eagle (DME) medium for 3 days (Fig. 5B). This capsule enlargement in the mutant cells was not observed when fungal cells were grown on noninducing media, such as YPD and yeast nitrogen base (YNB), suggesting that this phenotype is specific to capsule-inducible conditions (see Fig. S2 in the supplemental material). We also measured glucuronoxylomannan (GXM) secretion using two approaches. Using a Western blot assay with the GXM monoclonal antibody 18B7, we detected GXM components secreted into the medium after culturing cells for 0, 24, 48, and 72 h. Our results showed that the wild type secreted more GXM than the mutant, with a somewhat higher average molecular weight (Fig. 5C). We also quantified the amount of GXM secreted by purifying the secreted GXM in the medium supernatants after a 24-h incubation. We also found that the wild type secreted more GXM than the *cdc50Δ* mutant (Fig. 5D). Together, we conclude that the *cdc50Δ* mutant exhibited increased capsule size but reduced GXM secretion.

### Cdc50 is essential for *C. neoformans* to overcome the antifungal activity of macrophages.

**FIG 6:**
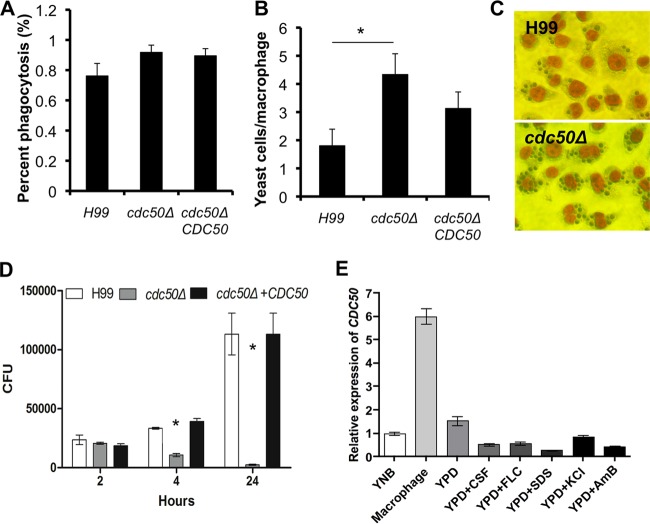
Cdc50 is required for *C. neoformans* to overcome the antifungal activity of macrophages. (A) The phagocytosis rate of *C. neoformans* in the J774 macrophage cell line was measured in 48-well plates. PBS-washed *C. neoformans* H99, *cdc50Δ*, and *cdc50Δ+CDC50* strains were added to the activated macrophages and incubated for 1.5 h at 37°C with 5% CO_2_. Cells were fixed with methanol and stained with Giemsa stain before counting. The percentage of macrophages containing internalized yeast cells was measured from over 100 macrophages. (B) In the same assay, the number of yeast cells in each macrophage was also calculated under an inverted microscope. The data were generated from counting over 500 macrophages. *, *P* < 0.01. (C) Typical field views of phagocytosis for each sample. (D) Intracellular proliferation of *C. neoformans* in the macrophage cell line. Cryptococcal strains were added to the activated macrophages and incubated for 2 h at 37°C with 5% CO_2_. Nonadherent extracellular yeast cells were then removed by washing with fresh DME medium. Numbers of fungal CFU from macrophage cultures after an additional 0, 2, or 22 h of incubation were used to determine intracellular proliferation and macrophage killing. The error bars indicate standard deviations for three experiments. *, *P* < 0.01. (E) RT-PCR analysis of *CDC50* gene expression under different culture conditions, including growth on YNB, YPD, YPD containing 8 µg/ml caspofungin (CSF), or YPD with 4 µg/ml fluconazole (FLC) and coculture with macrophages. The *GAPDH* gene was used as a reference. AmB, amphotericin B.

As it is a facultative intracellular pathogen, a key characteristic of *C. neoformans* is its ability to survive and replicate within the phagolysosome of macrophages (1, 26, 27). We investigated the potential role of Cdc50 in survival in macrophages. After coincubation of *C. neoformans* with macrophage cell line J774.16 for 1.5 h, macrophages were fixed with methanol and stained with Giemsa stain and the phagocytosis rate was determined by the number of yeast cells inside each macrophage. Our results showed that when cocultured with the wild-type strain H99, ~80% of macrophages contained yeast cells, with ~1.8 yeast cells on average inside each macrophage. These numbers increased to ~90% and ~4.3, respectively, when the *cdc50Δ* mutant was coincubated with macrophages, suggesting that Cdc50 helps cell resistance to phagocytosis (Fig. 6A to C). To further test macrophage killing of the fungal cells, *C. neoformans* and macrophages were coincubated for 2, 4, and 24 h; fungal cells were collected from macrophage cultures; and yeast survival rate was determined by CFU. Our results showed that the *cdc50Δ* mutant was hypersensitive to macrophage killing (Fig. 6D). To determine whether the killing of the *cdc50Δ* mutant cells was truly related to the presence of macrophages and not the medium, we cocultured fungal strains with YPD, fresh DME, fresh DME with 10% heat-killed fetal bovine serum (FBS), and the spent medium of J774.16 macrophage culture. Our results clearly showed that none of these culture conditions caused a significant CFU reduction in the *cdc50Δ* mutant, indicating that the killing of mutant cells is independent of the medium (see Fig. S3 in the supplemental material). These results demonstrate that *CDC50* is essential for fungal survival in macrophages.

### *CDC50* is highly expressed during interaction with macrophages.

To determine the expression pattern of the *CDC50* gene, we prepared *C. neoformans* RNAs from a range of culture conditions and measured gene expression using the quantitative real-time reverse transcription-PCR (qRT-PCR) method. Our data showed that the *CDC50* gene was highly induced when cocultured with the J774.16 macrophage cell line but that its expression was unaffected under other culture conditions, including treatment with caspofungin, fluconazole, or amphotericin B for 30 min, or under a variety of stress conditions, including SDS treatment and salt stress (Fig. 6E). Thus, *CDC50* expression is specifically induced during interaction with macrophages.

### Cdc50 regulates phosphatidylserine abundance on the cell surface.

In *S. cerevisiae*, *CDC50* encodes the β-subunit of the lipid flippase that directs the translocation of aminophospholipids, such as phosphatidylserine (PS), to maintain the asymmetry of the bilayer membrane structure (24). Our protein subcellular localization assay showed that *C. neoformans* Cdc50 localization is similar to that of Cdc50 proteins in other organisms (23, 24) (Fig. 3B), suggesting that Cdc50 in *C. neoformans* may also play a role in lipid translocation.

**FIG 7:**
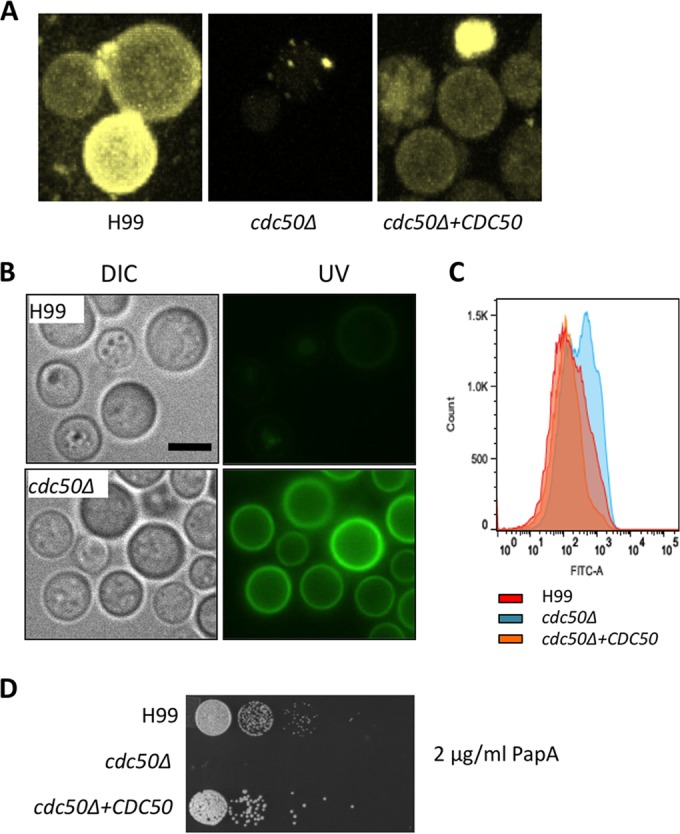
Loss of Cdc50 causes reduced phosphatidylserine (PS) uptake and increased PS levels on plasma membrane. (A) The PS uptake assay for H99, *cdc50Δ*, and *cdc50Δ+CDC50* strains was performed by coincubating fungal cells with 1 µmol NBD-PS. The fluorescent signal was photographed after a 30-min incubation at 30°C. Bar, 5 µm. (B) The fungal strains were coincubated with FITC-conjugated annexin V for 30 min before being fixed and observed by fluorescence microscopy. Bar, 5 µm. DIC, differential inference contrast. (C) The fluorescent signal of stained H99 and *cdc50*Δ cells was also determined by flow cytometry. One hundred thousand cells were counted for each sample. (D) The above strains were inoculated on YPD with 2 µg/ml papuamide A (PapA) and incubated at 30°C for 48 h.

The mutants of the Cdc50 homolog in mammalian cells have been reported to exhibit an aberrant exposure of endogenous aminophospholipids, including PS, on the cell surface (28). It has been suggested that PS exposure on the cell surface may serve as an “eat-me” signal for macrophages to recognize and internalize such cells (28). Therefore, the hypersensitivity of the *cdc50Δ* mutant to macrophage killing may be due not only to its defect in membrane integrity but also to increased exposure of PS on the cell surface. To test this hypothesis, we analyzed PS trafficking using 7-nitrobenz-2-oxa-1,3-diazol-4-yl (NBD)-labeled PS (NBD-PS). While wild-type H99 cells efficiently took up NBD-labeled PS, the *cdc50Δ* mutant displayed very weak NBD-PS binding and uptake, indicating a blockage of PS translocation, which is consistent with the function of Cdc50 as a subunit of the lipid flippase (Fig. 7A).

We also measured cell surface PS levels using fluorescein isothiocyanate (FITC)-conjugated annexin V staining (FITC-annexin), which efficiently binds to PS on the cell surface. Annexin V staining is commonly used to measure apoptosis because apoptotic cells have more PS on the outer plasma membrane. Our results showed that the FITC signal was much stronger in the mutant than in the wild-type strain (Fig. 7B). Signaling intensity quantification using flow cytometry confirmed the visual observations (Fig. 7C). In addition, we found that the *cdc50Δ* mutant is hypersensitive to papuamide A, a cytotoxic lipopeptide that kills fungal cells by binding to PS (Fig. 7D) (29, 30). These results indicate that the *cdc50Δ* cells have higher levels of PS exposure on the outer plasma membrane, which is consistent with observations in other eukaryotes (28). Because it has been reported that PS exposure causes macrophage recognition and macrophage killing in mammalian cells, this result may indicate that the increased sensitivity to killing by macrophages of the *C. neoformans* cdc50Δ mutant may be at least partially due to the high PS exposure in this mutant.

### Cdc50 is essential for fungal virulence.

**FIG 8:**
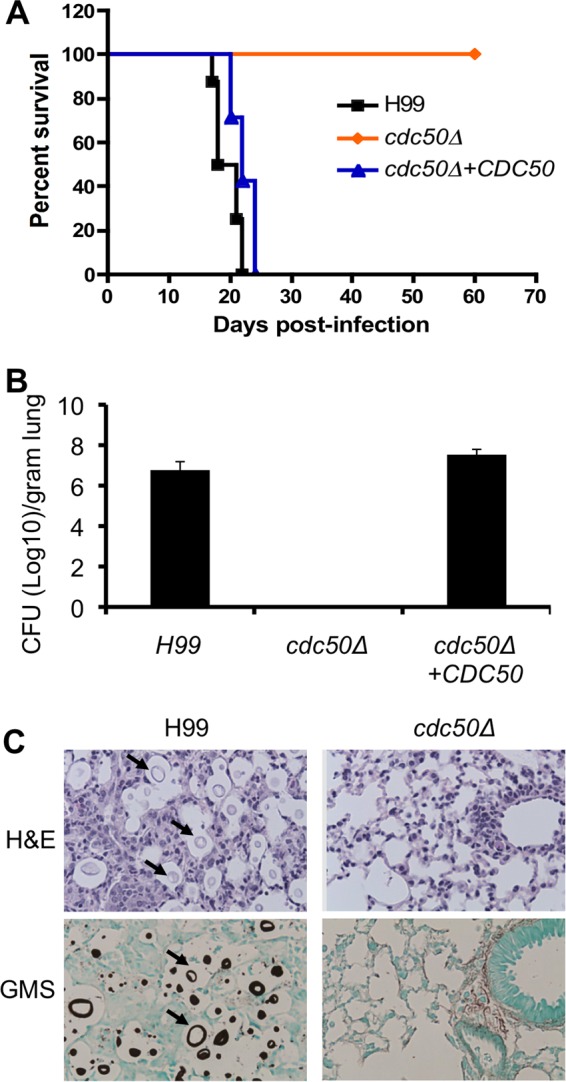
Cdc50 is required for virulence *in vivo*. (A) Survival curves for infected mice in a murine nasal inhalation model of systemic cryptococcosis. Female A/Jcr mice were inoculated via nasal inhalation with the (H99) *cdc50Δ* and *cdc50Δ+CDC50* strains. Mice were infected with 1 × 10^5^ yeast cells of each strain. (B) Fungal burden in infected lungs and brains was determined by CFU 7 days postinfection. (C) H&E- and GMS-stained slides were prepared from cross sections of infected lung at 7 days postinfection and visualized by light microscopy. The cryptococci are indicated by arrows.

Our results showing increased stress sensitivity, killing by macrophages, increased PS cell surface exposure, and altered virulence factor development in the *cdc50Δ* strain suggested that this mutant may affect *C. neoformans* virulence *in vivo*. To test this hypothesis, we used the wild type, the *cdc50Δ* mutant, and the complemented strain in a murine inhalation model of cryptococcosis. We observed that while mice infected by the wild-type strain died at around 20 days, as expected, mice infected by the *cdc50Δ* mutant remained healthy after 60 days postinoculation (Fig. 8A). Analysis of the fungal burden in the lungs and brains of *cdc50Δ* mutant-infected mice after 7 days postinfection showed that fungal cells were completely cleared in the lung (Fig. 8B). No fungal cells were observed in the sections of lungs infected by the mutant using either hematoxylin and eosin (H&E) or Grocott’s methenamine silver (GMS) staining, in contrast to the presence of abundant cryptococcal cells in the lung infected by the wild-type cells (Fig. 8C). The dramatic virulence attenuation and clearance of fungal cells from the lungs of mice infected by the *cdc50Δ* strain showed that the Cdc50 lipid flippase is essential for cryptococcal virulence *in vivo*.

## DISCUSSION

In this study, we showed that loss of lipid flippase component Cdc50 function leads to enhanced echinocandin and fluconazole sensitivity and increases *C. neoformans* sensitivity to several types of stress. Moreover, we found that the *CDC50* expression was specifically regulated by macrophage interaction and that the *cdc50Δ* mutant was highly sensitive to macrophage killing *in vitro*. Finally, Cdc50 was essential for fungal virulence in a murine model of systemic cryptococcosis. The detection of increased caspofungin uptake in the *cdc50Δ* mutant and increased PS exposure on the mutant cell surface help provide a mechanistic understanding of Cdc50 function in echinocandin resistance and fungal virulence.

Although β-1,3-glucan synthase is both functional and essential in *C. neoformans*, this fungus is highly resistant to echinocandin treatment (14). It is not currently understood why the efficacy of echinocandin therapy in *C. neoformans* is limited. Echinocandin drug resistance in *Candida* species is mostly due to the point mutations in β-1,3-glucan synthase subunits (9, 31). Because purified cryptococcal β-1,3-glucan synthase is sensitive to echinocandin *in vitro* (15), it is unlikely that this enzyme in *C. neoformans* naturally exists in an echinocandin-resistant mutant form, suggesting instead that the drug is not able to efficiently interact with the enzyme or disrupt the enzyme production *in vivo*. This conclusion is supported by our observation that a mutant that allows increased echinocandin entry into *C. neoformans* cells shows elevated echinocandin sensitivity.

Cdc50 homologs are best studied in *S. cerevisiae*, which contains three Cdc50 family members: Cdc50, Lem3, and Crf1. Cdc50 and Lem3 interact with P4-type ATPases Drs2 and Dnf1 to produce Cdc50-Drs2 and Lem3-Dnf1 complexes, respectively, which function as lipid flippases (17, 23, 32). Cdc50-like proteins contain two transmembrane domains and a large luminal loop (23), which is required for interacting with the transported phospholipids to translocate them through the membrane, thus maintaining the asymmetry of the bilayer lipid membrane (33). In *Cryptococcus*, Cdc50 is the only homolog of the Cdc50 gene family, and our study shows that it shares several properties with Cdc50 and Lem3 in *S. cerevisiae*, including its membrane localization and its role in aminophospholipid translocation. These similarities, together with the drug-sensitive and virulence-defective phenotype of the *cdc50Δ* mutant, suggest that cryptococcal Cdc50 also functions as part of a lipid flippase and that the function of this protein complex is necessary for both normal virulence and drug resistance.

The involvement of Cdc50 homologs in drug uptake has been reported previously. For instance, Cdc50A in human cells has been reported to play a role in the uptake of the anticancer drug perifosine (34). Also, Lem3 in *S. cerevisiae* has been shown to play an essential role in the uptake and potency of the alkylphosphocholine drugs edelfosine and miltefosine, which have been studied for treatment of protozoal and fungal diseases (35). However, in both cases, in contrast to *C. neoformans cdc50Δ* cells, the mutants lacking either *CDC50* or *LEM3* showed increased drug resistance due to blockage of drug uptake and translocation. In fact, *S. cerevisiae* diploids homozygous for *lem3Δ* have a higher caspofungin MIC than do the wild-type strains (36). Therefore, the drug-sensitive phenotype of the *C. neoformans cdc50Δ* mutant is unusual and may indicate a unique role of the lipid flippase in drug transport in this organism.

The detailed mechanisms of Cdc50-mediated caspofungin resistance will require further investigation. Studies in *Candida glabrata* have revealed a sphingolipid-dependent drug resistance mechanism where changes in membrane lipid composition lead to a tighter or looser interaction between various echinocandin-class drugs and their target, β-1,3-glucan synthase (36). Thus, it is possible that a change in membrane lipid distribution in the *cdc50Δ* mutant may alter the interaction of β-1,3-glucan synthase with caspofungin, resulting in improved drug efficacy. Another hypothesis is that caspofungin can be efficiently removed from the outer leaflet of the membrane, where it appears to act on glucan synthase in the wild-type *C. neoformans* cells by a lipid flippase-dependent process, and that this process is impaired in the mutant strain. Indeed, our caspofungin uptake assay using the BODIPY-labeled caspofungin showed a significantly increased intracellular caspofungin level in the mutant cells, suggesting that the mechanism of resistance is related to the caspofungin binding and internal caspofungin concentrations. In fact, lipid flippase is involved in the function of the ER and Golgi apparatus and has been reported to regulate the endocytic and exocytic recycling in *S. cerevisiae* (23, 32). Therefore, it is possible that the lipid flippase may contribute to echinocandin resistance in *C. neoformans* by either blocking drug penetration and interacting with the drug target or enhancing the drug exocytosis, leading to low intracellular drug concentrations.

We observed that Cdc50 also plays a role in the sensitivity of *C. neoformans* to azole drugs, such as fluconazole. Previous studies in *S. cerevisiae* have shown that Cdc50-mediated lipid flippase regulates the ergosterol distribution and trafficking in the Golgi network (37). Therefore, disruption of Cdc50 function could lead to altered ergosterol distribution on the membrane to cause lethality when treated with fluconazole. In agreement with this argument, it has been reported in *S. cerevisiae* that disruption of both *CDC50* and genes acting at late steps of the ergosterol biosynthesis pathway (*ERG2* and *ERG6*) results in synthetic lethality (38). Our screens also identified disruptions of three other genes encoding membrane proteins that have an elevated sensitivity to caspofungin. Two of them, *ERG3* and *ERG4*, encode enzymes involved in the ergosterol biosynthetic pathway. This discovery is consistent with our observation that many of our caspofungin-sensitive mutants are also sensitive to fluconazole.

Besides its role in drug resistance, our results have revealed the requirement of *C. neoformans* Cdc50 for fungal virulence both *in vitro* and *in vivo*. The *cdc50Δ* mutant showed a reduced growth rate at 37°C under some growth conditions *in vitro*, which may contribute to the virulence attenuation. Interestingly, *cdc50Δ* cells produced even larger capsules than did the wild type and caused no obvious difference in melanin production. Thus, it is not clear whether the virulence defect of the *cdc50Δ* mutant stems from altered production of these known cryptococcal virulence factors. Our results support another, non-mutually exclusive hypothesis for the role of Cdc50 in virulence: that Cdc50 activity is required for counteracting the antimicrobial activities of host macrophages. This hypothesis is supported by our findings that *CDC50* expression is specifically induced during interaction with macrophages, that *cdc50Δ* cells are engulfed by macrophages at a higher rate than the wild type and are more sensitive to macrophage killing *in vitro*, and that *cdc50Δ* mutants display higher levels of PS on the outer membrane, which may serve as a macrophage recognition signal, as demonstrated in other systems (28).

It is not clear whether the interaction of macrophages with PS exposed on the surface of cryptococcal cells is necessarily direct, as the presence of the large polysaccharide capsule in *C. neoformans* may interfere with this process. While we have no direct experimental evidence to address this question, studies in mammalian cells have shown that a direct interaction between macrophages and PS may be not required to trigger phagocytosis. In apoptotic cells where PS exposure on the cell surface acts as an “eat-me” signal to trigger phagocytosis, several macrophage-secreted proteins required for the recognition have been reported, such as milk fat-globule epidermal growth factor 8 (MFG-8), growth arrest-specific 6 (Gas6), and protein S (PROS), as reviewed in reference 39. These secreted proteins bind to PS to form a bridge between PS and macrophage cells during the recognition process. Thus, it is possible that macrophages are able to sense PS levels on the *C. neoformans* surface despite the presence of the capsule. One way to address this issue is to examine the phagocytosis of the *cdc50Δ* mutant in wild-type macrophages and macrophages lacking PS receptors. Furthermore, besides functioning as an “eat-me” signal, PS has recently been identified as a global immunosuppressive signal in efferocytosis, infectious disease, and cancer, as summarized in reference 40. Therefore, PS exposure on the cell surface may have multiple effects on *C. neoformans*-host interaction during infection, which will be interesting to investigate in the future.

In summary, we identified Cdc50, the only β-subunit homolog of lipid flippases in *C. neoformans*, as essential both for resistance to antifungal drugs in this organism and for virulence in a murine model of infection. These results reveal phospholipid translocation and trafficking as a new potential target for antifungal drug development.

## MATERIALS AND METHODS

### Strains and media.

An *Agrobacterium*-mediated mutagenesis library was created from *Cryptococcus neoformans* var. *grubii* (serotype A) strain H99. Two *Cryptococcus neoformans* gene deletion libraries were generated by Hiten Madhani at UCSF and purchased from the American Type Culture Collection (ATCC) and the Fungal Genetic Stock Center (FGSC), respectively. Other *C. neoformans* and *Saccharomyces cerevisiae* strains used in this study are listed in Table 2. In all growth assays, transformants were grown in nutrient-rich yeast extract-peptone-dextrose (YPD) medium at 30°C. After measurements were taken, cultures were stored at 4°C. Papuamide A was kindly provided by Kirk Gustafson at the National Cancer Institute. The GXM monoclonal antibody 18B7 was kindly provided by Arturo Casadevall at Johns Hopkins University. BODIPY-labeled caspofungin was synthesized in-house, while NBD-PS was purchased from Avanti. Caspofungin was provided by Merck. Other common medium preparation and growth conditions followed the instructions described previously (41, 42).

**TABLE 2:** Strains used in this study

Strain	Genotype	Reference or source
*C. neoformans*		
H99	*MAT*α	56
KN99**a**	MAT**a**	57
CUX178	Caspofungin-sensitive strain 11B6 from ATMT library	This study
CUX195	*MAT*α *mpt1Δ*::*NEO*	This study
CUX196	*MAT*α *cdc50*Δ::*NEO*	This study
CUX202	*MAT*α *cdc50Δ*::*NEO pHIS-GFP-CDC50-NAT*	This study
CUX208	*MAT*α *cdc50Δ*::*NEO CDC50-NAT*	This study
*S. cerevisiae*		
BY4741	*MAT***a** *his3Δ1 leu2Δ0 ura3Δ0*	ATCC deletion collection
YUX87	*MAT***a** *his3Δ1 leu2Δ0 ura3Δ0 lem3Δ*::*KanMX*	ATCC deletion collection
YUX89	*MAT***a** *his3Δ1 leu2Δ0 ura3Δ0 cdc50Δ*::*KanMX*	ATCC deletion collection

### Generation and screen of *Agrobacterium*-mediated mutagenesis library.

A random mutagenesis library was created using strains *C. neoformans* var. *grubii* H99 and *Agrobacterium tumefaciens* EHA105 as previously described (18). Briefly, cells were grown in various fungal-to-bacterial cell ratios. After 48 h, cultures were transferred to YPD with NAT and cefotaxime to select for *C. neoformans* strains with transfer DNA (T-DNA) insertions. Cells that grew on the selective medium were transferred to 96-well plates and grown to saturation for further screening.

To screen for mutants sensitive to caspofungin, mutagenesis libraries were activated by inoculating 5 µl stock cells to 96-well plates containing 150 µl YPD per well and incubated at 30°C. Once cultures were saturated, transformants were screened for sensitivity to 8 µg/ml of caspofungin. Ninety-six-well plates were prepared with 150 µl YPD plus 8 µg/ml of caspofungin. Five microliters of saturated cultures was inoculated into the corresponding wells. The optical density at 600 nm (OD_600_) was measured every 24 h, and growth in the presence of drug was compared to growth in YPD only. Transformants identified as having an increased sensitivity to drug compared to the wild type were selected for rescreening. After repeated screenings, sensitive mutants were selected for further analysis. Overnight cultures of transformants identified as sensitive to caspofungin were washed and diluted to a standardized OD_600_ of 1.0. Tenfold serial dilutions were prepared, and cultures were inoculated at different concentrations of caspofungin or fluconazole on YPD agar plates.

### Inverse PCR and gene identification.

Isolates identified as sensitive to caspofungin were crossed with strain KN99**a**. Cells from each culture were mixed and inoculated on MS and V8 mating media. Plates were incubated at 22.5°C in the dark for 10 days. Spores were dissected and inoculated on YPD-plus-NAT plates and incubated at 30°C for 3 days. Progenies were crossed with wild-type strain H99α to determine the mating type. Progenies with the **a** mating type were selected for phenotypic and genotypic analysis to determine whether the drug-sensitive phenotype of a progeny is cosegregated with the NAT marker.

For the mutants sensitive to caspofungin due to T-DNA insertion confirmed by cosegregation assay, their target genes were identified using the inverse PCR method as previously described (18) with minor adjustment. Briefly, genomic DNA was prepared and digested with ClaI, NcoI, NdeI, BglII, and XhoI for a total of five reactions. Digested fragments were self-ligated with T4 DNA ligase, the flanking regions of the NAT insert were amplified, and their sequences were determined. The location of the T-DNA insertion and subsequent gene disruption was determined using BLAST analysis of sequencing results with the *C. neoformans* genome database of the Broad Institute.

### *Cryptococcus*-macrophage interaction assay.

The macrophage-*Cryptococcus* interaction assay was done as previously described (43). Macrophage-like cell line J774 cells were cultured in DME medium with 10% heat-inactivated FBS at 37°C with 5% CO_2_. J774 cells (5 × 10^4^) in 0.5 ml fresh DME medium were added into each well of a 48-well culture plate and incubated at 37°C in 5% CO_2_ overnight. To activate macrophage cells, 50 units/ml gamma interferon (IFN-γ; Invitrogen) and 1 µg/ml lipopolysaccharide (LPS; Sigma) were added into each well. *C. neoformans* overnight cultures were washed with phosphate-buffered saline (PBS) twice and opsonized with 20% mouse complement. *Cryptococcus* cells (2 × 10^5^) were added into each well (yeast/J774 ratio, 4:1). To assess the phagocytosis rate, the cells were washed with PBS after a 1.5-h coincubation and fixed with methanol for 30 min. Giemsa stain was added to the wells at a 1:10 dilution, and the plates were incubated overnight at 4°C. Cells were washed once with PBS and analyzed using an inverted microscope. To assess intracellular proliferation of *C. neoformans*, nonadherent extracellular yeast cells were removed by washing with fresh DME medium after a 2-h coincubation and cultures were incubated for another 0, 2, and 22 h. At indicated time points, the medium in each well was replaced with distilled water (dH_2_O) to lyse macrophage cells for 30 min at room temperature. The lysate was spread on YPD plates, and CFU were counted to determine intracellular proliferation.

### qRT-PCR.

*Cryptococcus* cells from overnight culture or mating cultures were collected. Total RNA extraction and first-strand cDNA synthesis were followed with the protocol as described previously (42). Expression of *CDC50* and *GAPDH* was analyzed using SYBR Advantage QPCR premix reagents (Clontech). Gene expression levels were normalized using the endogenous control gene *GAPDH*, and the relative levels were determined using the comparative threshold cycle (*C_T_*) method (44). Real-time PCRs were performed using an Mx4000 QPCR system (Stratagene) as previously described (45).

### Generation of *cdc50*Δ mutant and its complemented strain.

The *cdc50*Δ mutants were generated by overlap PCR as previously described (46). The 5′ and 3′ regions of each *CDC50* gene were amplified from H99 genomic DNA with primers CX614/CX615 and CX616/CX617, respectively (see Table S1 in the supplemental material for primer sequences). The dominant selectable markers (Neo^r^) were amplified with the M13 primers (M13F and M13R) from plasmid pJAF1 (47). Each target gene replacement cassette was generated by overlap PCR with primers CX614/CX617 (see Table S1). Purified overlap PCR products were precipitated onto 10 µl gold microcarrier beads (0.6 µm; Bio-Rad), and H99 was biolistically transformed as described previously (48). Stable transformants were selected on YPD medium containing G418 (200 mg/liter). To screen for mutants of the *CDC50* gene, diagnostic PCR was performed by analyzing the 5′ junction of the disrupted mutant alleles with primers CX620 and JH8994 (see Table S1). Positive transformants identified by PCR screening were further confirmed by Southern blot analysis.

To generate complemented strains of *cdc50* mutants, a genomic DNA fragment that contains a 1.5-kb upstream promoter region, the *CDC50* ORF, and its 500-bp downstream region was amplified in a PCR using primers CX625/CX626. This PCR fragment was cloned into the plasmid pJAF13, which contains a NAT marker, by Infusion cloning (Clontech). The linearized plasmid was biolistically transformed in a *cdc50*Δ mutant strain to generate the complemented strain CUX208.

To generate a GFP-tagged strain, the *CDC50* full-length cDNA was amplified with primers CX647/CX648 and cloned into the BamHI/NotI sites of a vector containing the *Cryptococcus* actin promoter (49) and a GFP epitope, to generate a plasmid that contains the CDC50-GFP fusion. The above plasmids were biolistically transformed into the wild-type H99 strain to generate strain CUX202, which would express Cdc50-GFP.

### Assays for melanin, capsule production, and stress response.

Melanin production was assayed by inoculating *C. neoformans* strains into 2 ml YPD liquid medium and incubating them overnight at 30°C. Five microliters of each overnight culture with serial dilutions was placed on l-3,4-dihydroxyphenylalanine (l-DOPA) agar medium. The agar plates were incubated at 30°C or 37°C for 2 days, and pigmentation of fungal colonies was assessed. To examine capsule production, yeast cells were inoculated on Dulbecco modified Eagle (DME) medium. The DME medium plates were incubated at 37°C for 3 days before capsule size was visualized by adding a drop of India ink to the cell suspensions and observed on an Olympus AX70 microscope (Melville, NY). The relative capsule size was calculated by dividing capsule size with the whole-cell size (capsule and cell size). The average and standard deviation from at least 50 cells were calculated for each condition tested. Electromobility of secreted GXM from H99 and the *cdc50Δ* mutant was detected in a Western blot assay as described in reference 50. Secreted total polysaccharides were purified from a 500-ml YPD culture of each strain using the cetyltrimethylammonium bromide (CTAB) precipitation method as described previously (51). The amount of the total GXM was determined by the phenol sulfuric method. The two-pair *t* test method was used to determine the statistical significance of the difference between samples.

To assay for stress responses, yeast cells from overnight cultures were washed, resuspended, and serially diluted (1:10) in dH_2_O and spotted (5 µl) on YPD agar plates containing 1.0 M KCl for osmotic shock, 2.5 mM H_2_O_2_ for oxidative stress, and 1 mM NaNO_2_ for nitrosative stress as previously described (43, 52). To test cell integrity, cells were also spotted on YPD agar plates containing 0.03% SDS, 0.5% Congo red, or 250 µg/ml calcofluor white (CFW) and incubated for 2 days at 30°C.

### Cellular staining assays. (i) BODIPY-caspofungin localization assay.

*C. neoformans* and *S. cerevisiae* overnight cultures were reinoculated in YPD and cultured for 4 h. Fresh cultures were washed with PBS, and BODIPY-fluorescently labeled caspofungin was added at a final concentration of 5 µM and incubated for 30 min. Cell suspensions were then observed under a fluorescence microscope for cellular localization of the fluorescent signal. Fluorescent signal intensity for 100,000 cells was also quantified by flow cytometry.

### (ii) Annexin V assay.

*Cryptococcus* strains were cultured in YPD for 48 h. Cells were harvested and washed in binding buffer (10 mM HEPES-NaOH, pH 7.4, 140 mM NaCl, and 2.5 mM CaCl_2_) and resuspended in 1 ml binding buffer containing 5 µl FITC-conjugated annexin V (Life Technologies, Inc.) as described previously (53). After being incubated for 1 h at 30°C with shaking, cells were fixed by 3.7% formaldehyde for 10 min at 37°C and then washed in PBS containing 1% formaldehyde before being observed under fluorescence microscopy. Fluorescent signal intensity was also quantified by flow cytometry.

### (iii) NBD-PS uptake assay.

*C. neoformans* cells from overnight cultures were washed in NaCl buffer (0.7 M NaCl, 0.5 mM MgCl_2_, 35 mM potassium phosphate, pH 6.8) twice and treated with 15 U/ml lyticase (Sigma) for 2 h at 30°C as previously described (54). Cells were incubated in NaCl buffer with 100 µmol NBD-PS for 30 min at 30°C and then washed 3 times with NaCl buffer containing 4% bovine serum albumin (BSA) before being fixed with 3.7% formaldehyde for 10 min at 37°C. Fixed cells were washed with NaCl buffer and observed under fluorescence microscopy or quantified using flow cytometry.

### (iv) ER-Tracker Red staining.

*C. neoformans* overnight cultures were washed in Hanks balanced salt solution (HBSS) buffer (1.26 mM CaCl_2_, 5.33 mM KCl, 0.44 mM KH_2_PO_4_, 0.5 mM MgCl_2_⋅6H_2_O, 0.44 mM MgSO_4_⋅7H_2_O, 138 mM NaCl, 4 mM NaHCO_3_, 0.3 mM Na_2_HPO_4_, and 5.6 mM d-glucose) twice before being fixed with 3.7% formaldehyde for 2 to 5 min at 30°C. Fixed cells were washed with HBSS buffer 3 times and stained with 1 µmol ER-Tracker (Life Technologies, Inc.) for 30 min at 30°C before being observed by fluorescence microscopy (Nikon).

### Virulence studies *in vivo* and fungal burden in infected organs.

Groups of 10 female A/Jcr mice (NCI-Frederick) were intranasally infected with 10^5^ yeast cells of each strain as previously described (41, 55). Over the course of the experiments, animals that appeared moribund or in pain were sacrificed by CO_2_ inhalation. Survival data from the murine experiments were statistically analyzed between paired groups using the log rank test of the PRISM program 4.0 (GraphPad Software, San Diego, CA). *P* values of <0.001 were considered significant.

Infected animals were sacrificed at the endpoint of the experiment according to the Rutgers IACUC-approved animal protocol. For mice infected by the *cdc50Δ* mutant strain, the experiment was terminated 60 days postinfection. To compare fungal burdens, lungs and brains from mice infected by H99, the *cdc50Δ* mutant, or its complemented strain were isolated 7 days postinfection. Infected lungs and brains were isolated and homogenized in 1× PBS buffer using a homogenizer. Resuspensions were diluted, and 100 µl of each dilution was spread on YPD medium; fungal colonies were counted after 3 days of incubation at 30°C. The isolated animal tissues were also fixed in 10% formalin solution and sent to the Rutgers histopathological core facility for section preparation and staining with hematoxylin and eosin (H&E) and Grocott’s methenamine silver (GMS) stain.

## SUPPLEMENTAL MATERIAL

Figure S1  Cdc50 in *C. neoformans*, but not in *S. cerevisiae*, plays a negative role in caspofungin internalization. (A) Cultures of *C. neoformans* H99, *cdc50Δ*, and *cdc50Δ+CDC50* strains were coincubated with 5 µmol BODIPY-labeled caspofungin for 30 min at 30°C. The fluorescent signal of fungal cells was detected by fluorescence microscopy. (B) *C. neoformans* survival rate was determined by CFU for the above strains treated with 5 µmol BODIPY-caspofungin for 30 min at 30°C. (C) Cultures of *S. cerevisiae* strain BY4742 and its *lem3Δ* and *cdc50Δ* mutants were coincubated with 5 µmol BODIPY-labeled caspofungin for 30 min at 30°C. The fluorescent signal of fungal cells was detected by fluorescence microscopy. Download Figure S1, PDF file, 2.3 MB

Figure S2  The *C. neoformans* cells lacking *CDC50* produced normal capsule under noninducing conditions for capsule. *C. neoformans* H99, *cdc50Δ*, and *cdc50Δ+CDC50* strains and the *CDC50* overexpression strain (*P_HIS_-GFP-CDC50*) were cultured on YNB and YPD for 3 days, respectively. Capsule production of these cells on YNB (A) and YPD (B) was visualized by India ink staining. (C) Capsule sizes were measured for the above strains cultured in either YNB or YPD medium from over 100 cells for each condition. Download Figure S2, PDF file, 1.8 MB

Figure S3  The *C. neoformans* cells lacking *CDC50* had normal growth rates on different media. *C. neoformans* H99, *cdc50Δ*, and *cdc50Δ+CDC50* strains were cultured on YPD, DME, or DME with 10% FBS or on macrophage spent medium. Numbers of CFU were used to determine live cell numbers after incubation for 2, 4, and 24 h in different media. Download Figure S3, PDF file, 0.3 MB

Table S1  Primers used in this study. Table S1, DOCX file, 0.1 MB
