# Supplementary figures and images for: Lipid Flippase Subunit Cdc50 Mediates Drug Resistance and Virulence in Cryptococcus neoformans
Source: mBio. 2016 May 10;7(3):e00478-16. doi: 10.1128/mBio.00478-16 (PMC4959666; doi:10.1128/mBio.00478-16)

Fig. S1

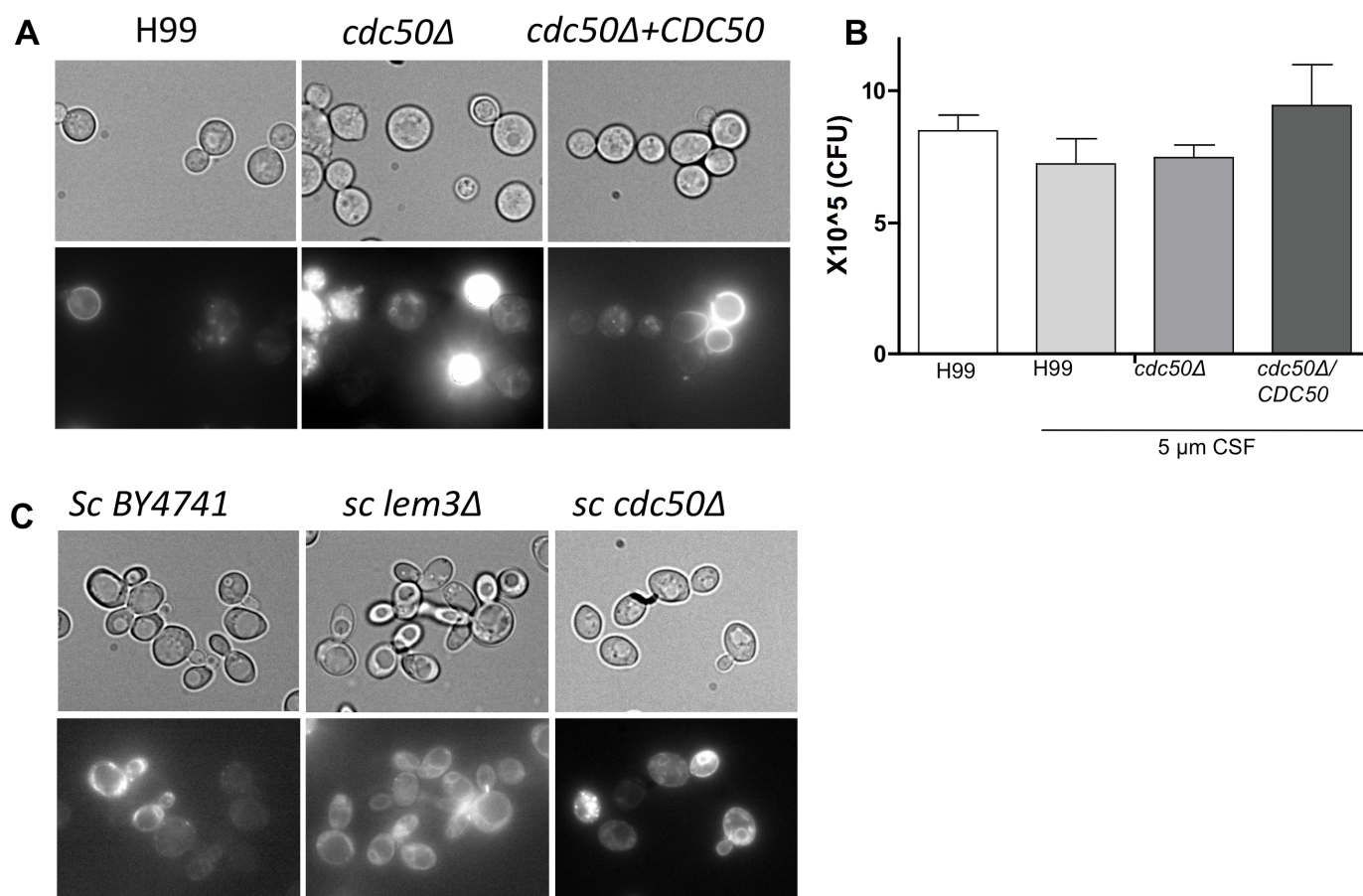

Supplement: Figure S1 — Cdc50 in C. neoformans, but not in S. cerevisiae, plays a negative role in caspofungin internalization. (A) Cultures of C. neoformans H99, cdc50Δ, and cdc50Δ+CDC50 strains were coincubated with 5 µmol BODIPY-labeled caspofungin for 30 min at 30°C. The fluorescent signal of fungal cells was detected by fluorescence microscopy. (B) C. neoformans survival rate was determined by CFU for the above strains treated with 5 µmol BODIPY-caspofungin for 30 min at 30°C. (C) Cultures of S. cerevisiae strain BY4742 and its lem3Δ and cdc50Δ mutants were coincubated with 5 µmol BODIPY-labeled caspofungin for 30 min at 30°C. The fluorescent signal of fungal cells was detected by fluorescence microscopy. Download [file mbo002162814sf1.pdf]

Fig. S2

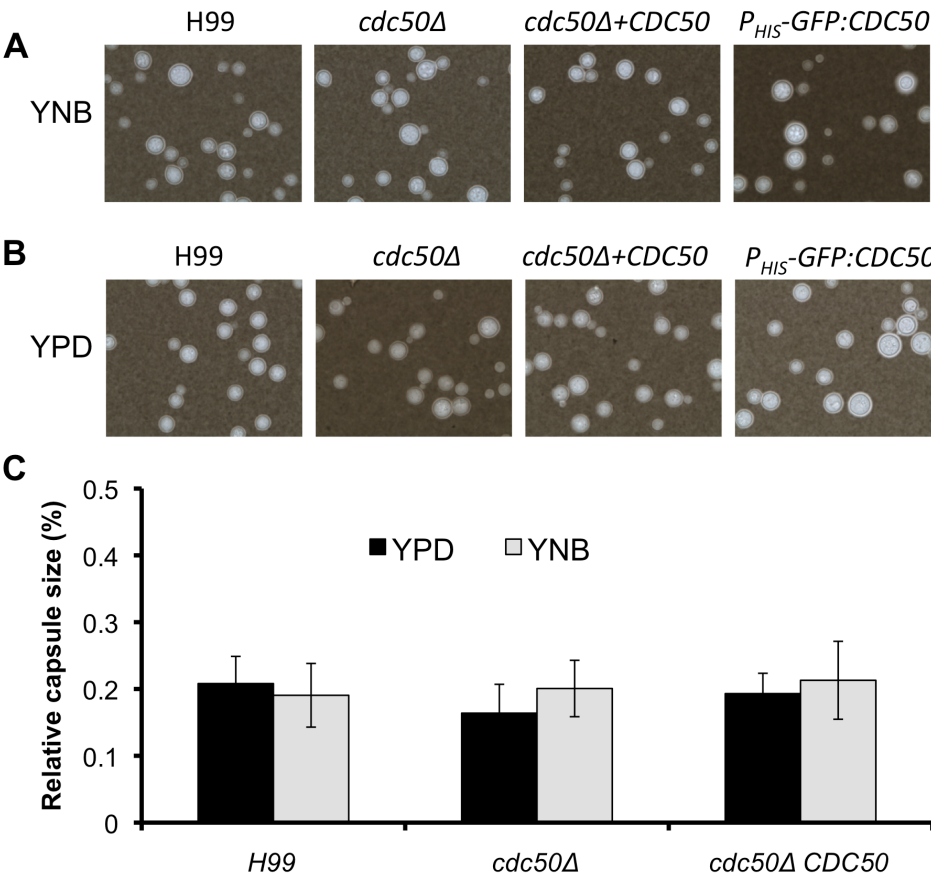

Supplement: Figure S2 — The C. neoformans cells lacking CDC50 produced normal capsule under noninducing conditions for capsule. C. neoformans H99, cdc50Δ, and cdc50Δ+CDC50 strains and the CDC50 overexpression strain (PHIS-GFP-CDC50) were cultured on YNB and YPD for 3 days, respectively. Capsule production of these cells on YNB (A) and YPD (B) was visualized by India ink staining. (C) Capsule sizes were measured for the above strains cultured in either YNB or YPD medium from over 100 cells for each condition. Download [file mbo002162814sf2.pdf]

Fig. S3

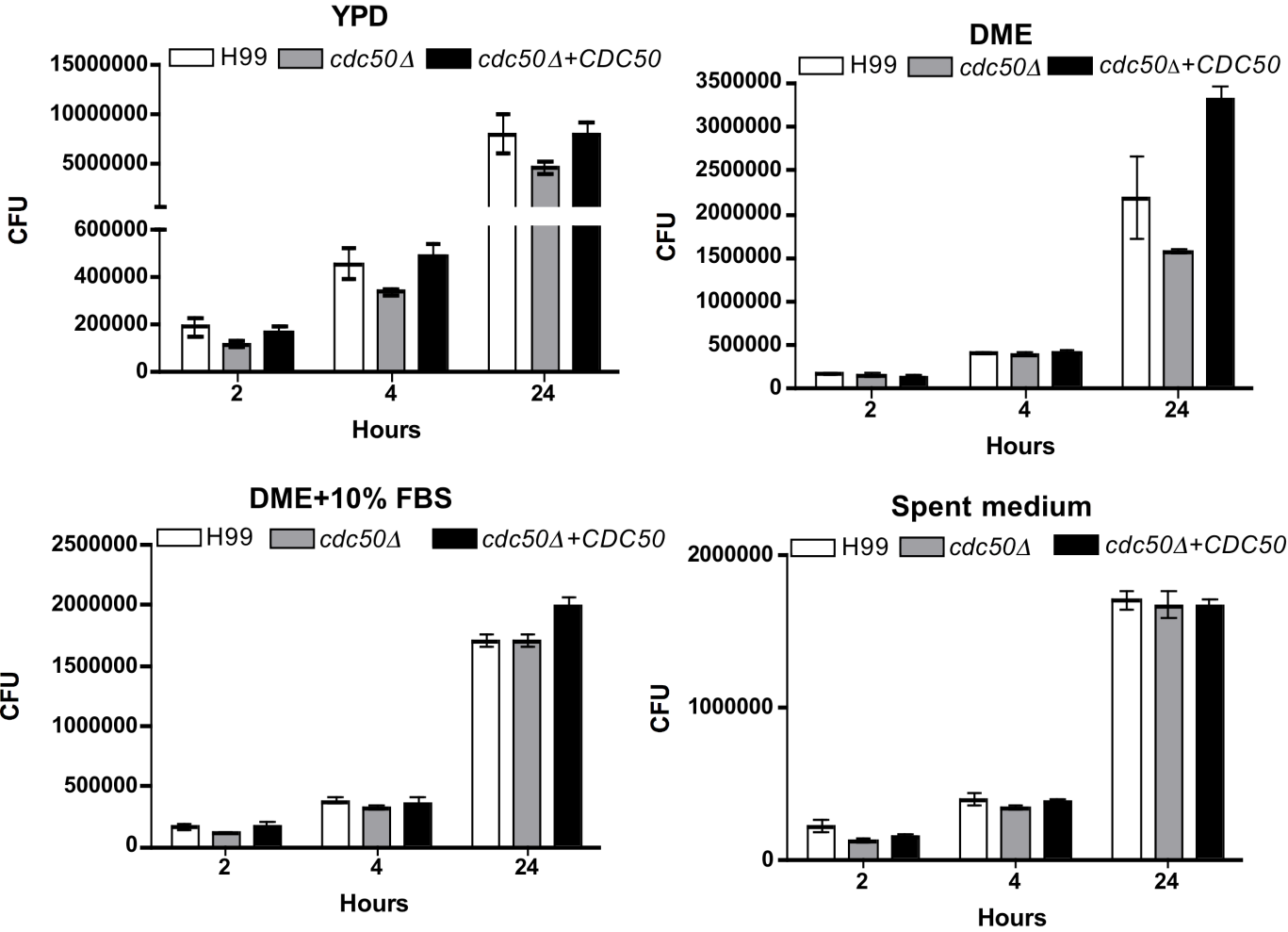

Supplement: Figure S3 — The C. neoformans cells lacking CDC50 had normal growth rates on different media. C. neoformans H99, cdc50Δ, and cdc50Δ+CDC50 strains were cultured on YPD, DME, or DME with 10% FBS or on macrophage spent medium. Numbers of CFU were used to determine live cell numbers after incubation for 2, 4, and 24 h in different media. Download [file mbo002162814sf3.pdf]
