# Supplementary material for: Lipid Flippase Subunit Cdc50 Mediates Drug Resistance and Virulence in Cryptococcus neoformans
Source: mBio. 2016 May 10;7(3):e00478-16. doi: 10.1128/mBio.00478-16 (PMC4959666; doi:10.1128/mBio.00478-16)
Supplement: Table S1 — Primers used in this study. [file mbo002162814st1.docx]

Table 2 Primers used in this study

| Primers | Sequences (5’-3’) | References/Note |
| --- | --- | --- |
| CX5 | GTAAAACGACGGCCAG | M13F |
| CX6 | CAGGAAACAGCTATGAC | M13R |
| CX607 | TCTCTGCGAAACAAAAACACC | MPT1 F1 |
| CX608 | CTGGCCGTCGTTTTACACAATAACAAAGACAACAACAGAC | MPT1 R1 |
| CX609 | GTCATAGCTGTTTCCTGGCCATTGCGCTTCTACTATCTAC | MPT1 F2 |
| CX610 | CGCGCCCCAACAATTATCCATCAA | MPT1 R2 |
| CX611 | GATCGGGTGGAGGGCGGAAGTGT | MPT1 F3 |
| CX612 | TGTTGCGGCTGGAAAGGAGTATGG | MPT1 R3 |
| CX613 | AACGGGGTCCTTGAGAGTGATA | MPT1 F4 |
| CX614 | GCGGTAGATCGGGAAAATGTGGTT | CDC50 F1 |
| CX615 | CTGGCCGTCGTTTTACGGTGGGATGGTGGACGGTGAA | CDC50 R1 |
| CX616 | GTCATAGCTGTTTCCTGTTGGTTTGTTCTTTTTGATGATTT | CDC50 F2 |
| CX617 | GCGCGTATGATGATTCCGATTGG | CDC50 R2 |
| CX618 | CCGCTAGGTGTGAGATTGAGTTTG | CDC50 F3 |
| CX619 | GAATCCGTCCACATAGCCGTTAGG | CDC50 R3 |
| CX620 | CAAACGGCGAGACTGGTAAAGACA | CDC50 F4 |
| CX625 | ATCCACTAGTTCTAGACCATGTCCGAACCTGTCACCT | CDC50 complement F5 |
| CX626 | TGGCGGCCGCTCTAGACCTCCTGGATGGTGGCATGC | CDC50 complement R5 |
| CX627 | **CGAGCTGTACGGATCCATG**GCCATATTCAACAGGAAGC | CDC50 GFP F6 |
| CX628 | **CGTTACTAGTGGATCCTTA**TAATCCATTTGCGTTGGGCT | CDC50 GFP R6 |
| CX647 | GAATTCCCGGGGATCCGAATGGCCATATTCAACAGGAAGC | CDC50 F7 |
| CX648 | GCAGGTCGACGGATCCTTATAATCCATTTGCGTTGGGCTG | CDC50 R7 |
| **CX752** | TAGACGGCCATTACGGCCATGGCCATATTCAACAGGAAGCC | CDC50-Forward |
| **CX753** | CCTTTGGCCGAGGCGGCCCTTAATCCATTTGCGTTGGGCTGG | CDC50-Reverse |
| CX953 | AGGTGTGAGATTGAGTTTGAGG | CDC50F QPCR |
| CX954 | GATATAAGTGAGGGTGGAGGC | CDC50R QPCR |
| JH8994 | TGTGGATGCTGGCGGAGGATA | For mutant screen |
| JH8956 | AACAGTTGCGCAGCCTGAATG | For inverse PCR |
| JH8957 | AGAGGCGGTTTGCGTATTGG | For inverse PCR |
